# Antiphospholipid reactivity against cardiolipin metabolites occurring during endothelial cell apoptosis

**DOI:** 10.1186/ar2091

**Published:** 2006-12-06

**Authors:** Cristiano Alessandri, Maurizio Sorice, Michele Bombardieri, Paola Conigliaro, Agostina Longo, Tina Garofalo, Valeria Manganelli, Fabrizio Conti, Mauro Degli Esposti, Guido Valesini

**Affiliations:** 1 Dipartimento di Clinica e Terepia Medica, Cattedra e Divisione di Reumatologia, Università La Sapienza, viale del Policlinico 155, Roma, 00161, Italy; 2 Dipartimento di Medicina Sperimentale e Patologia, Università La Sapienza, viale Regina Elena 324, Roma, 00161, Italy; 3 Laboratrorio di Medicina Sperimentale e Patologia Ambientale, Università La Sapienza, viale dell'Elettronica, Rieti, 02100, Italy; 4 Rheumatology Department, Kings College, Guy's Hospital, St Thomas Street, London, SE1 9RT, UK; 5 Faculty of Life Sciences, University of Manchester, Oxford Road, Manchester, M13 9PT, UK

## Abstract

We have recently shown that cardiolipin (CL) and its metabolites move from mitochondria to other cellular membranes during death receptor-mediated apoptosis. In this study, we investigate the immunoreactivity to CL derivatives occurring during endothelial apoptosis in patients with antiphospholipid syndrome (APS) and systemic lupus erythematosus (SLE). We compared the serum immunoreactivity to CL with that of its derivatives monolysocardiolipin (MCL), dilysocardiolipin (DCL), and hydrocardiolipin (HCL) by means of both enzyme-linked immunosorbent assay and thin-layer chromatography (TLC) immunostaining. In addition, we investigated the composition of phospholipid extracts from the plasma membrane of apoptotic endothelial cells and the binding of patients' sera to the surface of the same cells by using high-performance TLC and immunofluorescence analysis. The average reactivity to MCL was comparable with that of CL and significantly higher than that for DCL and HCL in patients studied, both in the presence or in the absence of beta_2_-glycoprotein I. Of relevance for the pathogenic role of these autoantibodies, immunoglobulin G from patients' sera showed an increased focal reactivity with the plasma membrane of endothelial cells undergoing apoptosis. Interestingly, the phospholipid analysis of these light membrane fractions showed an accumulation of both CL and MCL. Our results demonstrated that a critical number of acyl chains in CL derivatives is important for the binding of antiphospholipid antibodies and that MCL is an antigenic target with immunoreactivity comparable with CL in APS and SLE. Our finding also suggests a link between apoptotic perturbation of CL metabolism and the production of these antibodies.

## Introduction

Patients with antiphospholipid syndrome (APS) show high levels of circulating antiphospholipid antibodies (aPLs) and are prone to arterial and venous thrombosis, recurrent abortions, and/or foetal loss [1-3]. aPLs are heterogeneous antibodies binding to phospholipids, proteins, or phospholipid-protein complexes. Historically, cardiolipin (CL) was used as an antigen for aPL determination [4]. In 1990, different reports described the requirement of beta_2_-glycoprotein I (β_2_-GPI) for the binding of anticardiolipin antibodies (aCLs) in solid-phase immunoassays [5-7]. Although β_2_-GPI represents the best target antigen in the pathogenesis of APS, other phospholipid-binding proteins have been described as phospholipid cofactors [8-12]. Moreover, it has been reported that in infectious diseases aCL recognises CL in the absence of β_2_-GPI [13-16].

CL is a unique anionic phospholipid composed of two phosphate groups and four fatty acid chains and represents the most unsaturated (susceptible of oxidation) lipid of the body [17,18]. In particular, the degree of unsaturation of the acyl chains in CL influences the binding of β_2_-GPI to CL, whereas (hydro)peroxidation of CL has been shown to be essential for enhancing the binding of aPLs [19]. Although CL is predominantly associated with the inner mitochondrial membrane (where CL synthase is located), its rapid re-modelling into the highly unsaturated species that are most common in adult tissues (for example, tetra-linoleyl-CL) occurs in other membranes. CL re-modelling, in fact, involves relocation to the outer mitochondrial membrane, as well as to extra-mitochondrial compartments [17,18], with rapid de-acylation into mono- and di-lysocardiolipin (with three and two acyl chains, respectively). These metabolites are transported to the endoplasmic reticulum (ER) for efficient re-acylation into the mature forms of CL found in mitochondria in a process that seems to be facilitated by lipid transfer proteins like Bid [20]. Consistent with such a multi-organelle cycle of CL re-modelling, we have recently shown that CL is exposed on the plasma membrane (PM) of cells undergoing apoptosis induced by death receptors like Fas and tumour necrosis factor-alpha (TNF-α) [21,22]. This translocation onto the cell surface implies a leakage of CL (and/or of its metabolites) from the normal re-modelling cycle [20], probably as a consequence of an apoptosis-mediated increase of ER and secretory membranes. This leak might well represent an *in vivo *trigger for the generation of aCL [21,22]. Interestingly, mass spectroscopy analysis has demonstrated an early degradation of mitochondrial CL into its immediate metabolite, monolysocardiolipin (MCL), during Fas-induced apoptosis [23]. This finding has been subsequently confirmed in human pro-monocytic U937 cells [22].

The recent data have increased our attention on the role of CL metabolites and, in general CL acylation, on the generation and properties of aCL, a subject that has been analysed by a limited number of studies so far [24,25]. In the present study, we demonstrate that the number of acyl chains in CL derivatives is important not only for the binding of β_2_-GPI, but also for the generation of epitopes of 'pure' aCL for specific CL metabolites like MCL. In addition, we show that CL and its key derivative, MCL, re-locate to the PM of human umbilical vein endothelial cells (HUVECs) undergoing apoptosis. We suggest that aCL and antimonolysocardiolipin antibody (aMCL) might derive from alteration in the metabolism of CL as a possible consequence of enhanced apoptosis.

## Materials and methods

### Patients

Twenty-eight immunoglobulin G (IgG) aCL-positive outpatients were enrolled after clinical referral to the Division of Rheumatology of the University of Rome 'La Sapienza.' All patients were positive for medium-to-high levels of IgG aCL according to our standard enzyme-linked immunosorbent assay (ELISA) (β_2_-GPI-dependent) 89 GPL, range 40 to 120). Eighteen patients had APS according to the Sapporo criteria [3] primary (*n *= 6) or secondary (*n *= 12) to systemic lupus erythematosus (SLE), and 10 patients had SLE fulfilling American College of Rheumatology criteria [26]. The clinical and serological features of the patients are summarised in Table 1. We enrolled 24 healthy subjects as controls (13 female and 11 male; mean age 34 years, range 22 to 52 years). Finally, to establish whether aPL reactivity from patients with infections was different than that from patients with APS and SLE, we selected three sera from 37 hepatitis C virus (HCV) patients previously deemed positive for IgG aCL by ELISA and thin-layer chromatography (TLC) immunostaining [27]. None of the healthy subjects or the selected HCV patients experienced arterial or venous thrombosis or recurrent foetal loss. After informed consent was obtained, each subject underwent peripheral blood sample collection and the sera were then stored at -20°C until assayed.

### Materials

CL (bovine heart) was obtained from Sigma-Aldrich (St. Louis, MO, USA). MCL, dilysocardiolipin (DCL), and hydrogenated ('reduced') CL (HCL) were obtained from Avanti Polar Lipids, Inc. (Alabaster, AL, USA). TLC was performed as previously described [28] to assess the purity of the phospholipid preparations (data not shown). Human β_2_-GPI was obtained from Chemicon International (Temecula, CA, USA).

In addition to human sera, the following antibodies were used: rabbit polyclonal anti-human β_2_-GPI (Chemicon International), mouse monoclonal anti-transferrin receptor (CD71; BD Pharmingen, San Diego, CA, USA), goat polyclonal anti-voltage-dependent anion channel-1 (VDAC-1)/porin (N18; Santa Cruz Biotechnology, Inc., Santa Cruz, CA, USA), and mouse monoclonal anti-subunit IV of cytochrome c oxidase (COX-IV) (Molecular Probes, now part of Invitrogen Corporation, Carlsbad, CA, USA); alkaline phosphatase-conjugated secondary antibodies (goat anti-human and mouse anti-rabbit IgG) were purchased from Sigma-Aldrich.

Human IgG fractions were first isolated with 33% ammonium sulphate fractionation from plasma of patients with APS and from healthy donors as previously described [21,22]. Protein concentration was measured with the method of Lowry and colleagues [29], and the purity of the IgG preparations was confirmed by SDS-PAGE.

### ELISA for aPLs

Briefly, pure phospholipids (50 μg/ml) in ethanol were used to coat microtitre plates by incubation at 4°C overnight. In this way, oxidation of phospholipids occurs during the coating process as previously reported [19]. After four washes with phosphate-buffered saline (PBS), plates were blocked for 1 hour at room temperature with PBS containing 10% foetal calf serum (PBS-F). After four washes with PBS-F, plates were incubated for 90 minutes at room temperature with sera samples diluted at 1:50 or human IgG (100 μl of concentrated solutions of 4.8 mg/ml) in PBS-F. Titration of aCL/aMCL-positive sera was performed by serial dilution (1:25 to 1:1,000) in PBS-F by using measurements in triplicate. Moreover, a rabbit polyclonal anti-β_2_-GPI was used to detect the levels of β_2_-GPI bound to lipids. After four washes with PBS-F, the plates were incubated for 90 minutes at room temperature with secondary anti-human IgG and anti-rabbit IgG (Sigma-Aldrich) diluted to 1:1,000 in PBS-F; after multiple washes, immunoreactivity was developed using the alkaline phosphatase substrate (paranitrophenyl phosphate in ethanolamine). The enzyme reaction was evaluated from the absorbance at 405 nm in a plate reader. To account for the different molecular weights of CL derivatives, we performed ELISA with lipids coated to the plate at the same molarity. Finally, to perform β_2_-GPI-independent ELISA, 1% bovine serum albumin (BSA) or 0.25% gelatine was used in the blocking and washing steps. All assays were performed at least in duplicate, and the non-specific binding was evaluated by subtracting the absorbance of non-specific binding of each serum in wells without antigens.

### Absorption test

To investigate the specificity of the assay, competitive inhibition tests were performed according to technique described previously [30,31]. Briefly, serum samples (1:50 in PBS containing 1% BSA) were pre-incubated for 60 minutes at 37°C with increasing amounts of CL or its derivatives dried onto the surface of glass tubes. Subsequently, the tubes were centrifuged (10,000 *g *for 30 minutes) and the supernatants were tested for reactivity toward CL derivatives.

### Immunostaining on TLC plates for aPLs

The immunostaining of TLC plates (Merck, Darmstadt, Germany) was performed as described previously [28], using 2.5 μg of CL, MCL, DCL, and HCL. IgG fractions (2.4 mg/ml) from both APS sera (which had been deemed aCL-positive by standard ELISA screening) and normal human sera were diluted 1:100 or 1:1,000 in PBS containing 0.5% (wt/vol) gelatine. Parallel blots were processed without primary antibody or without antigen as control for non-specific reactivity.

### Western blotting

BSA, gelatine, and human β_2_-GPI were run on 12% SDS-PAGE and electro-transferred to nitrocellulose membrane (Bio-Rad Laboratories, Inc., Hercules, CA, USA). Membranes were blocked with PBS containing 5% defatted dry milk and then probed with rabbit polyclonal anti-β_2_-GPI antibodies. Antibody binding was detected using alkaline phosphatase-conjugated secondary antibodies and visualised with a kit containing chromogenic alkaline phosphatase substrate (Bio-Rad Laboratories, Inc.) according to manufacturer's instructions. The reaction was stopped by washing in distilled water.

### Indirect immunofluorescence of cells

Cellular indirect immunofluorescence was carried out to analyse CL expression on the PM of HUVECs, which were isolated by collagenase perfusion from normal-term umbilical cord veins as described previously [32]. Cells were cultured in M-199 medium (Sigma-Aldrich) supplemented with 20% foetal calf serum (FCS), 2 mM L-glutamine, 100 U/ml penicillin, and 100 μg/ml streptomycin at 37°C in a humidified atmosphere of 5% CO_2_. HUVECs were grown to 60% to 70% confluence, transferred at 5 × 10^6 ^cells per well on glass coverslips, either untreated or treated with 20 ng/ml TNF-α and 10 μg/ml cycloheximide for 16 hours [33,34], and fixed in PBS containing 4% formaldehyde for 1 hour at 4°C. Apoptosis was evaluated by morphologic analysis and by propidium iodide staining according to Nicoletti and colleagues [35]. After three washes with PBS, cells were incubated for 1 hour at 4°C with purified human IgG from APS patients (aCL- and aMCL-positive) in PBS containing 1% BSA. Alternatively, after previous absorption with CL or MCL as reported above, cells were incubated for 1 hour at 4°C with purified human IgG from APS patients. Fluorescein isothiocyanate-conjugated anti-human IgG (γ-chain specific; Sigma-Aldrich) were then added and incubated at 4°C for 30 minutes. After a washing with PBS, fluorescence was analysed with an Olympus U RFL microscope (Olympus, Tokyo, Japan). In parallel experiments, cells were directly stained before formaldehyde fixation. Alternatively, cells were processed for a second formaldehyde fixation immediately after the incubation with purified IgG and before the addition of the secondary antibody. Neither fixation procedure affected the staining on the cell surface [21].

### Phospholipid analysis on light membrane fractions

Subcellular fractions were isolated from HUVECs as previously reported [22]. Briefly, control untreated cells or cells treated with 20 ng/ml TNF-α and 10 μg/ml cycloheximide for 16 hours were rinsed with cold isolation buffer (0.25 M mannitol, 1 mM EDTA, 10 mM K-HEPES, 0.2% BSA, pH 7.4) containing a cocktail of protease inhibitors (Sigma-Aldrich), resuspended in 1 ml of the same buffer, and homogenised vigorously. After a brief centrifugation at 600 *g *in the cold isolation buffer, pellet and supernatant were combined, rehomogenised, and centrifuged at 800 *g *for 10 minutes at 4°C. The pellet was discarded and the supernatant was further centrifuged at 10,000 *g *for 10 minutes at 4°C. The pellets (P10) were washed three times with assay buffer (0.12 M mannitol, 0.08 M KCl, 1 mM EDTA, 20 mM K-HEPES, pH 7.4, containing a cocktail of protease inhibitors) and then resuspended in the same buffer. The supernatant was further centrifuged at 100,000 *g *for 1 hour at 4°C to obtain the cytosolic supernatant (S100) and the light membrane pellet (P100). The latter was dissolved in assay buffer, normalised for protein content by Bio-Rad protein assay (Bio-Rad Laboratories, Inc.), split into two aliquots, and analysed for phospholipid composition by TLC and for the presence of mitochondrial and endosomal contaminants by Western blotting. Phospholipids were extracted according to the technique described by Folch and colleagues [36] and separated by TLC by using high-performance TLC (HPTLC) silica gel 60 (10 × 10) plates (Merck). Chromatography was performed in chloroform/methanol/acetic acid/water (100:75:7:4) (vol/vol/vol/vol). After exposure to iodide vapours, the bands comigrating with the CL and MCL standard were scraped, eluted from silica with chloroform/methanol (2:1) (vol/vol), and dried under nitrogen. Phospholipids were run using methanol/chloroform/NH_3 _(7:13:1) and stained with copper acetate. Alternatively, light membranes were diluted with assay buffer containing protease inhibitors and adjusted to a final protein concentration of 0.5 to 1 mg/ml with concentrated SDS sample buffer. Protein samples were separated by SDS-PAGE and blotted in PBS containing 0.05% Tween-20 with the following antibodies to exclude the presence of mitochondrial contaminants: mouse monoclonal anti-transferrin receptor (CD71), goat polyclonal anti-VDAC-1/porin, and mouse monoclonal anti-COX-IV. Blots were visualised by chemiluminescence reaction by using the ECL Western detection system (Amersham Biosciences, now part of GE Healthcare, Little Chalfont, Buckinghamshire, UK). Protein loading was evaluated by india ink staining [37].

### Statistical analysis of data

Statistical analysis of data was carried out using Mann-Whitney's *U *test for comparison of means between different groups of subjects. Wilcoxon paired test was used to compare differences between aCL derivative profiles in each group of patients and controls. Correlation analysis was carried out by the Spearman test. *P *less than 0.05 was considered statistically significant.

## Results

### Binding of aPLs to CL and its derivatives by ELISA

**Figure 1:**
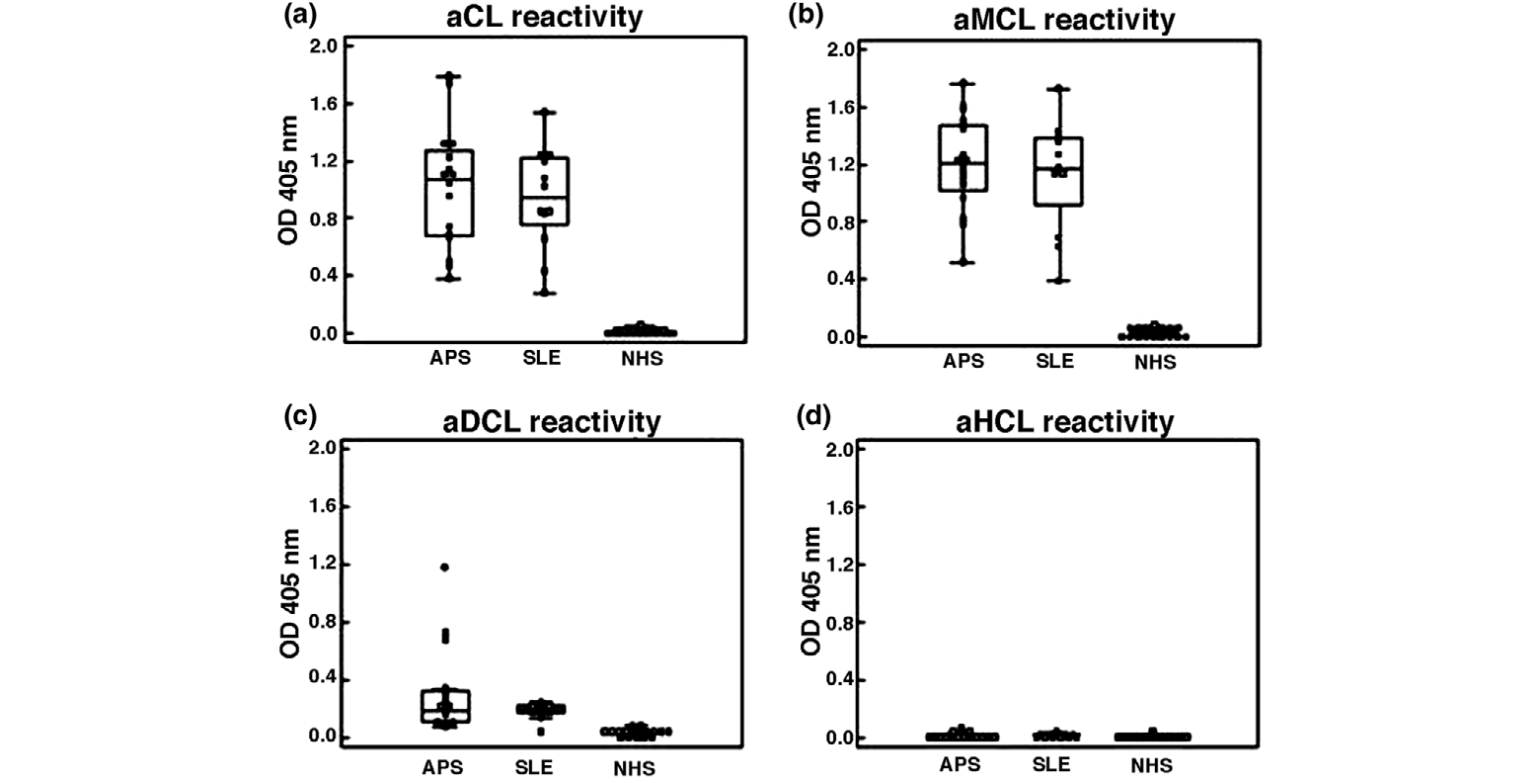
Box-and-whisker plots of autoantibodies binding to cardiolipin (CL) and its derivatives. Median, quartiles, range, and possibly extreme values are shown. NHS indicates healthy donors. **(a) **anticardiolipin reactivity; **(b) **antimonolysocardiolipin reactivity; **(c) **antidilysocardiolipin reactivity; **(d) **antihydrocardiolipin reactivity. The average immunoreactivities to CL and monolysocardiolipin were significantly higher than those for dilysocardiolipin and hydrocardiolipin, both in antiphospholipid syndrome (APS) (*P *< 0.0001) and systemic lupus erythematosus (SLE) patients (*P *< 0.001) **(a, b)**. aCL, anticardiolipin antibody; aDCL, antidilysocardiolipin antibody; aHCL, antihydrocardiolipin antibody; aMCL, antimonolysocardiolipin antibody; OD, optical density.

**Figure 2:**
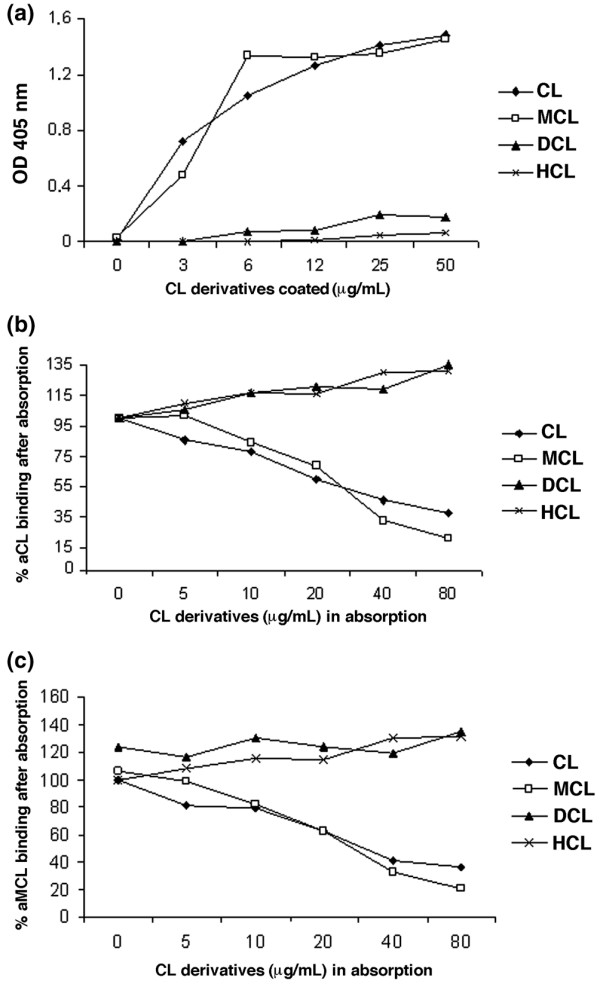
Dilution and absorption tests. **(a) **Immunoglobulin G binding of selected sera positive for both anticardiolipin antibody (aCL) and antimonolysocardiolipin antibody (aMCL) was enhanced by increasing the concentration of cardiolipin (CL) derivatives coated on the enzyme-linked immunosorbent assay (ELISA) plate wells. To further characterise the antigen specificity, three aCL/aMCL-positive sera were tested in ELISA after pre-absorption with increasing concentrations of CL derivatives. Pre-absorption with CL and monolysocardiolipin (MCL) significantly decreased the binding of aCL to CL **(b) **and of aMCL to MCL **(c)**, respectively. No significant competitions were observed with dilysocardiolipin (DCL) and hydrocardiolipin (HCL) pre-absorption concerning the binding of both aCL and aMCL sera **(b, c)**. Each data point represents the mean of triplicate determinations. OD, optical density.

The levels of serum antibodies reacting with CL, MCL, DCL, and HCL in patients and controls were quantified by ELISA, and the overall data are shown in Figure 1. Similar results were obtained when purified human IgG were used instead of sera (data not shown). The average immunoreactivity to CL and MCL was significantly higher than that for DCL and HCL, both in APS (*P *< 0.0001) and SLE patients (*P *< 0.001) (Figure 1). In addition, the same reactivity profile was observed when CL derivatives were coated onto plates at the same molarity, even if under these conditions the amount (weight) of coated lipids may have been slightly different. All three patients with HCV positive for aCL IgG were also positive for aMCL. Thus, our results indicated that, at least in solid-phase immunoassays, a key derivative of CL such as MCL represents a lipid easily recognised by sera autoantibodies. To study this novel observation in more detail, we performed titrations with representative sera showing strong reactivity for both CL and MCL (Figure 2a). The concentration dependence of the antibody titre indicated that binding to MCL saturated as efficiently as that to CL, whereas reactivity toward both DCL and HCL remained very low and did not saturate at all (Figure 2a). To further characterise the IgG specificity of binding, three sera positive for both aCL and aMCL were tested in ELISA after absorption with increasing amounts of CL and its derivatives. As shown in Figure 2b, pre-absorption with both CL and MCL significantly decreased the binding of aCL to CL. Similar results were observed when binding of aMCL to MCL was analysed after absorption with CL and MCL (Figure 2c). In contrast, when sera were pre-absorbed with either DCL or HCL, no significant competition in the binding of both aCL and aMCL was observed (Figure 2b,c). These findings suggest that CL and MCL present identical epitopes, or at least overlapping epitopes.

### β_2_-GPI dependence of immunoreactivity to CL and its derivatives

All the aCL/aMCL-positive sera of patients with APS and SLE were found to react also with β_2_-GPI as detected by standard ELISA (data not shown). Conversely, none of the controls (healthy subjects and patients with HCV) was positive for anti-β_2_-GPI.

**Figure 3:**
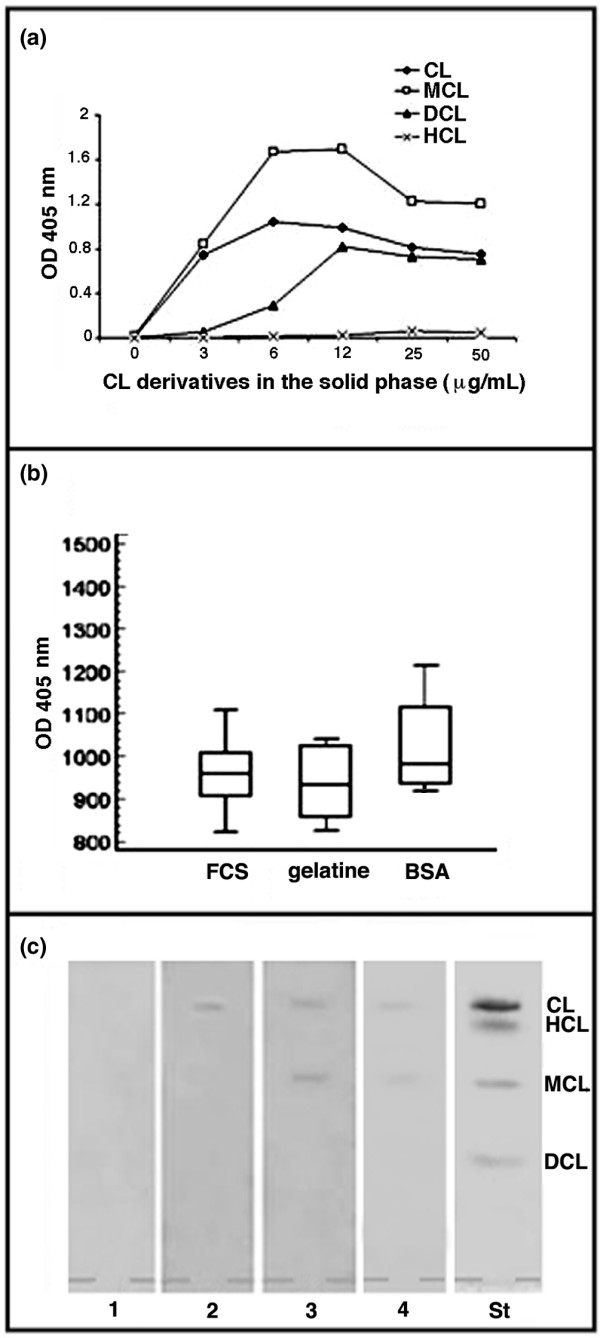
Beta_2_-glycoprotein I (β_2_-GPI) dependence of immunoreactivity to cardiolipin (CL) derivatives. **(a) **Binding of β_2_-GPI to CL and its derivatives. Intriguingly, β_2_-GPI showed significant binding to CL, monolysocardiolipin (MCL), and dilysocardiolipin (DCL), which was comparable with that for CL at the highest concentration tested. Each data point represents the mean of triplicate determinations. **(b) **Box-and-whisker plot of antimonolysocardiolipin antibody (aMCL) binding in six antiphospholipid syndrome (APS) patients. Median, quartiles, range, and possibly extreme values are shown. The blocking and the washing steps were performed with foetal calf serum (FCS) (10%) in phosphate-buffered saline (PBS)-Tween-20 to provide the β_2_-GPI or with bovine serum albumin (BSA) (1%) or gelatine (0.5%) to avoid the presence of β_2_-GPI, which is commonly associated with FCS. No significant difference of aMCL reactivity was observed with different blocking solutions. **(c) **Thin-layer chromatography (TLC) immunostaining analysis of: lane 1: normal serum, diluted 1:100 in PBS/0.5% gelatine; lane 2: control positive aCL serum, diluted 1:100 in PBS/0.5% gelatine; lane 3: APS serum positive for both aCL and aMCL, diluted 1:100 in PBS/0.5% gelatine; lane 4: APS serum positive for both aCL and aMCL, diluted 1:1,000 in PBS/0.5% gelatine. No sera showed reactivity against DCL and hydrocardiolipin (HCL). The results in lanes 3 and 4 are representative of five different APS patients. OD, optical density; St, standard phospholipid visualisation of lipids (cardiolipin, hydrocardiolipin, monolysocardiolipin, and dilysocardiolipin) by iodide vapours.

We observed that the isolated β_2_-GPI protein could bind to MCL other than CL (Figure 3a). Intriguingly, the protein also showed a significant binding to DCL, with an affinity that appeared to be comparable with that for CL. So, although β_2_-GPI attached itself to both MCL and CL, it equally reacted with DCL *in vitro *(Figure 3a), whereas APS sera displayed only low reactivity toward DCL (Figures 1 and 2). In this respect, it is interesting to note that the reactivity of SLE and APS sera toward other negatively charged lipids such as lyso(bis)phosphatidic acid (LBPA) [27,38] could be mediated by the relatively non-specific interaction that serum proteins like β_2_-GPI may have with chemically different phospholipids sharing a negative charge.

To determine whether aCL/aMCL binds directly to lipids, we performed ELISA in the absence of serum proteins (for example, FCS) or in the presence of other proteins like BSA or gelatine. Clearly, patients' autoantibodies retained high reactivity toward both CL and MCL in the absence of FCS and irrespective of the exogenous proteins used for blocking the plate matrix (Figure 3b). To exclude the possibility that BSA or gelatine was contaminated with the serum protein β_2_-GPI that avidly binds CL and its derivatives (Figure 3a), we performed Western blot analysis with a specific anti-β_2_-GPI antibody. The results did not show detectable levels of β_2_-GPI in the samples of BSA or gelatine used in the experiments (data not shown).

In another approach, we tested the immunoreactivity of aCL/aMCL-positive sera toward pure lipids separated by TLC in the complete absence of exogenous proteins that might interfere with the autoantibody reactivity with phospholipids. TLC immunostaining of five representative aCL/aMCL-positive sera showed strong reactivity to a combination of CL and MCL (Figure 3c). No sera showed reactivity against DCL and HCL. Next, we performed experiments to assess the possible contribution of β_2_-GPI toward aCL and aMCL reactivity in TLC immunostaining. Considering that β_2_-GPI is normally present in concentrations of approximately 200 μg/ml in human sera, we performed immunostaining on TLC with sera that had been diluted to 1:1,000. With such a dilution, β_2_-GPI levels would fall below the minimum requirement (>0.5 μg/ml) for allowing binding of aCL to anionic phospholipids [39]. As previously demonstrated [40,41], TLC immunostaining showed a reactivity with CL and MCL up to a dilution of 1:1,000 (Figure 3c, lane 4), thereby excluding a major contribution of serum proteins to the observed immunoreactions to CL derivatives.

### Binding of aCL and aMCL to HUVECs undergoing apoptosis

**Figure 4:**
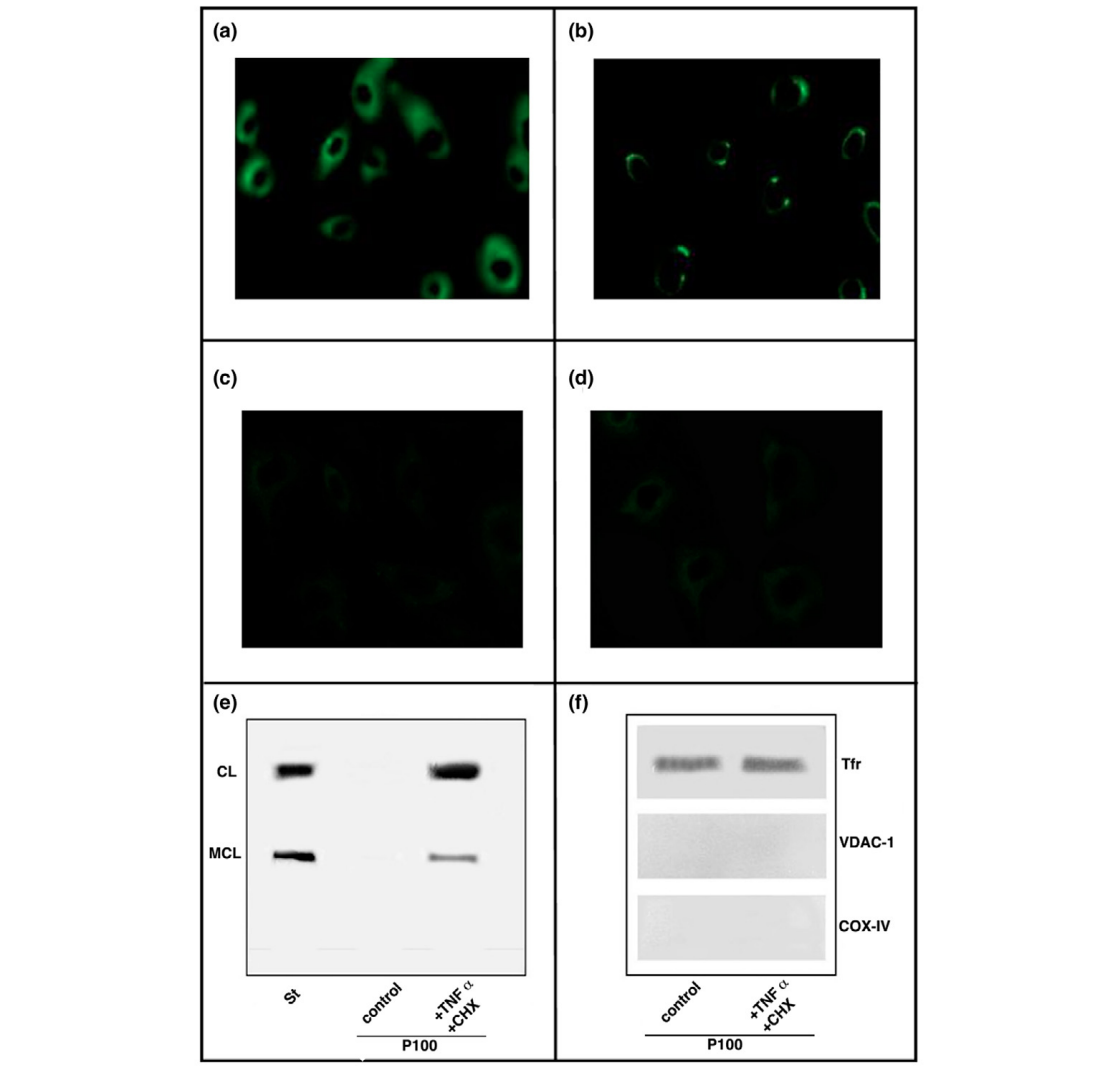
Binding of aCL antibodies and aMCL to HUVECs undergoing apoptosis. **(a) **Immunofluorescence of untreated HUVECs. The staining of cells with the human immunoglobulin G (IgG) fractions from antiphospholipid syndrome (APS) sera was followed by fluorescein isothiocyanate-conjugated secondary antibody. Cells showed a fairly low and disperse immunolabelling. **(b) **Immunofluorescence of apoptotic HUVECs. The staining appears uneven and focalised in regions over the plasma membrane. Immunofluorescence of untreated HUVECs after previous absorption with both **(c) **CL and **(d) **MCL. The staining of cells with the human IgG fractions from APS sera was very low. Images were collected at 512 × 512 pixels, and results are representative of three repeats. **(e) **Light membrane pellets from either apoptotic or non-apoptotic HUVECs were analysed for phospholipid composition by thin-layer chromatography. Strong bands corresponding to CL and MCL were identified only in the membrane fractions of apoptotic HUVECs. **(f) **Western blot analysis of protein samples from light membranes showed strong reactivity against the membrane protein marker transferrin receptor (Tfr), with the virtual absence of mitochondrial (VDAC-1/porin and COX-IV) contaminants. CHX, cycloheximide; COX-IV, subunit IV of cytochrome c oxidase; St, standard phospholipid visualisation of lipids (cardiolipin, hydrocardiolipin, monolysocardiolipin, and dilysocardiolipin) by iodide vapours; TNF-α, tumour necrosis factor-alpha; VCAC-1, voltage-dependent anion channel-1.

To evaluate whether apoptosis may represent a possible trigger for autoantibody production toward CL and MCL, human IgG fractions from APS patients were used to analyse the distribution pattern of CL (and MCL also) on the surface of apoptotic HUVECs. Reactivity showed specificity for CL and MCL, given that no significant reactions were detected by TLC immunostaining between IgG fractions and other phospholipids such as phosphatidylserine, phosphatidylinositol, and LBPA. No antinuclear reactivity of IgG fractions was observed by standard indirect immunofluorescence on Hep2 cells. To promote apoptosis, we used treatment with TNF-α plus cycloheximide under conditions that were previously shown to induce high levels of endothelial cell death [33,34]. Fluorescence microscopy analysis revealed that human IgG fractions displayed a stronger binding to the surface of HUVECs undergoing apoptosis as compared with untreated cells (Figure 4a,b). The enhanced staining onto the PM appeared uneven, indicating that the reactivity was concentrated in localised areas on or near the cell surface. Most likely, these areas coincided with PM patches where active membrane traffic and re-modelling were most intense (for example, blebbing precursors, which subsequently give rise to apoptotic bodies) (Figure 4b). A virtual absence of immunolabelling was observed across internal membranes, suggesting that intracellular CL was somehow shielded from autoantibody reactivity. Interestingly, a very low staining was observed after previous absorption with CL or MCL in both untreated cells (Figure 4c and 4d, respectively) or cells treated with TNF-α plus cycloheximide (not shown). As a negative control, human IgG from healthy subjects showed negligible surface staining of HUVECs, either untreated or after TNF-α and cycloheximide treatment (not shown).

To further investigate the above observations, we isolated light membrane fractions containing PMs of apoptotic and untreated HUVECs. Phospholipids were extracted and the presence of CL and MCL was verified by HPTLC (Figure 4e). Strong bands corresponding to CL and MCL were identified only in the membrane fractions of apoptotic cells. The PM enrichment of the light membrane fractions was confirmed by Western blot analysis, which showed strong reactivity against the membrane protein marker transferrin receptor, with the virtual absence of mitochondrial (VDAC-1/porin and COX-IV) contaminants (Figure 4f). Hence, the results obtained with HUVECs undergoing apoptosis were similar to those reported previously with U937 cells [21,22] and indicated that CL and MCL become surface-exposed and much more accessible to autoantibody recognition after induction of apoptosis.

## Discussion

In this study, we report for the first time that sera from APS and SLE patients display strong reactivity toward MCL, a key metabolite of CL. MCL is the immediate product of CL degradation, a process that has been shown to occur either in mitochondria (both membranes) as part of the re-modelling cycle of the mature lipid or in lysosomes [20,42]. CL is among the most resistant phospholipids toward phospholipase A2 hydrolysis in membranes of healthy cells. However, significant levels of MCL exist in healthy tissues and increase after stimulation of physiological apoptotic pathways such as Fas/FasL, which are relevant to white blood cells and immunological response [22,23]. In this context, our finding of autoantibodies that cross-react with CL and MCL could provide a pathologically relevant link between the metabolic cycle and membrane traffic of CL and apoptosis. Indeed, CL is known to undergo degradation (including peroxidation) during many pathways of cell death [42].

Recent findings have suggested that CL and its metabolites may be transported by apoptosis regulator proteins like Bid, especially after caspase cleavage [23,43]. On the other hand, we recently demonstrated that CL becomes exposed onto the PM of myelomonocytic cells undergoing apoptosis *in vitro*, suggesting that intracellular traffic of CL may enhance CL immunogenicity *in vivo *[21] as previously described for other autoantigens [44]. Of relevance to this issue, Casciola-Rosen and coworkers [45] have shown aCL binding to surface blebs of apoptotic cells, which would be consistent with the clustering of aCL immunostaining in focal surface regions that we detected in apoptotic endothelial cells. This indicates that cells undergoing apoptosis expose CL on their surface in segregated membrane regions that could enhance the binding of circulating autoantibodies. In this view, we consider that our previous findings of CL reactivity on the cell surface of apoptotic cells [21] could reflect a combined recognition of exposed CL and MCL. Indeed, our original findings in the present work suggest a prominent role of both CL and MCL in eliciting a pathological autoimmune reactivity in APS and related conditions. Moreover, we demonstrated for the first time the relocation of CL and MCL to the PM of apoptotic HUVECs, which are largely involved in the pathogenesis of APS. In contrast, in our extensive mass spectrometry (MS) studies of membrane lipids and their changes after the induction of apoptosis, we detected very low levels of MS signature ions for DCL species in the PM of apoptotic cells, suggesting that in most cells DCL is present in trace amounts with respect to CL and MCL, most likely reflecting a combination of rapid re-modelling turnover in the ER and lysosomal degradation in by-products of CL catabolism [22]. These findings might explain the low DCL reactivity that we found in sera of both APS and SLE patients.

Our data also demonstrate that the chemistry of the acyl chains of CL is important not only for the binding to β_2_-GPI, but also for the intrinsic immunogenicity of the CL molecule. We also demonstrate here that the serum protein β_2_-GPI shows a differential binding to CL derivatives (MCL > CL > DCL), which, although similar, does not entirely match the reactivity exhibited by SLE and APS sera. In fact, by using both ELISA and TLC immunostaining, we found that sera can also bind with high efficiency to MCL and CL in the absence of β_2_-GPI. Thus, our data support the notion that aCL and anti-β_2_-GPI represent two distinct populations of antibodies [40] with overlapping but not coincident antigenic recognition. In this respect, pathogenic human monoclonal antibodies that bind phospholipids in the absence of β_2_-GPI have been described [46,47]. Likewise, the binding of plasmatic β_2_-GPI to CL and MCL on the cell surface after apoptotic stimuli is possible. Thus, we cannot exclude the possibility that aPLs may also be directed to this phospholipid-protein complex.

After an early report showing different reactivity of various CL derivatives with Wassermann antibody [48], only a few investigations have been published on the role of the acyl chains of CL in binding of aPLs [24,25,49-51]. Notably, Qamar and co-workers [49] suggested a potential role of the fatty acyl chains in the binding of autoantibodies to lysophosphatidylethanolamine. Another study concluded that aPL binding as monitored with an ELISA system depends on the fatty acid moiety of phospholipids but is also influenced by the nature of their polar head and phosphodiester groups [24]. Interestingly, the authors of this work suggested that *in vivo *aPLs may bind to intermediate ('transition') membrane phospholipids, which could expose their fatty acid chains that contributed to antibody recognition. In accordance with recent insights into the role of CL and its re-modelling in apoptosis [23,42,43], we propose that both CL and MCL are the 'transition' membrane lipids suggested earlier [24]. In agreement with this possibility, Berger and colleagues [25] suggested that the number of acyl chains in CL may represent a significant factor in the binding of aPLs. However, they detected a significant decrease of binding to MCL in 12 SLE aCL-positive sera [25]. The discrepancy with our data may be due to the selection of patients based on their high reactivity to CL or to the small number of patients tested as well as to the different dilution of the sera [25]. More recently, antibodies to lysophosphatidylcholine (LPC) have been identified indicating that LPC may also contribute to the antigenicity of oxidised low-density lipoproteins [49,50]. Moreover, a strong correlation between antibodies directed to LPC and CL was found [50]. It is noteworthy that LPC is a by-product of CL re-modelling, PC being the predominant acyl donor for MCL during re-modelling [18]. On the other hand, it has also been suggested that CL, after oxidation, could be hydrolysed by serum phospholipase, thus creating molecular structures similar to either LPC or MCL [50]. Interestingly, phospholipase A2 activity is enhanced in both autoimmune and inflammatory diseases [51], as well as during apoptosis [20]. Consistent with our study, it is thus possible that CL hydrolysis of one fatty acid chain to yield MCL enhances CL antigenicity. In addition, MCL has hybrid properties between a diacyl-lipid (bilayer-forming) and a lyso-lipid (micelle-forming), which may physically facilitate autoantibody reactivity. In contrast, there are fundamental physico-chemical reasons why autoantibodies fail to bind to DCL because this lipid has an unusually large and hydrophilic polar head with respect to any other (di-acyl) phospholipid. DCL may have as many as five free hydroxyl groups in its polar head, some of which are likely to produce intramolecular hydrogen bonds that would change the geometry and overall structure of the lipid. Then, in the likely event of partial peroxidation of the acyl chains, additional hydrogen bonds could be formed between the polar head groups and the oxygenated fatty acids, producing large changes that alter the overall structure of the molecule. Such changes will strongly modify the docking properties and on/off constants of lipid binding to the cognate hydrophobic pockets in serum immunoglobulins (or other CL-interacting proteins). In the case of APS serum proteins, their binding to CL is critically determined by the conformation and chemistry of the acyl chains, as demonstrated here by the lack of binding to the hydrogenated CL analogue, which shows baseline levels comparable with those of DCL. Whereas MCL could maintain the overall structural determinants that enable the recognition of CL, the further loss of an acyl chain in DCL destroys the binding affinity because of the large structural alterations that distinguish this lipid from its related metabolites and, overall, from most other di-acyl lipids.

## Conclusion

In this study, we describe a high reactivity of APS and SLE sera to MCL, the immediate degradation product of mitochondrial CL. We propose a model in which apoptosis or inflammatory processes enhance the hydrolysis (and membrane traffic) of CL into MCL, which can then escape the metabolic cycle of lipid re-modelling. MCL can then become exposed to the cell surface and consequently to the immune system, either directly or through the interaction with a plasma protein such as β_2_-GPI. Interestingly, these events may occur *in vivo *in cells directly involved in the pathogenesis of APS, such as monocytic and endothelial cells, as demonstrated by us in *in vitro *experiments. Hence, a deranged process of CL metabolism could stimulate autoantibody reactivity by the synergistic binding to MCL.

## Abbreviations

aCL = anticardiolipin antibody; aMCL = antimonolysocardiolipin antibody; aPL = antiphospholipid antibody; APS = antiphospholipid syndrome; β_2_-GPI = beta_2_-glycoprotein I; BSA = bovine serum albumin; CL = cardiolipin; COX-IV = subunit IV of cytochrome c oxidase; DCL = dilysocardiolipin; ELISA = enzyme-linked immunosorbent assay; ER = endoplasmic reticulum; FCS = foetal calf serum; HCL = hydrocardiolipin; HCV = hepatitis C virus; HPTLC = high-performance thin-layer chromatography; HUVEC = human umbilical vein endothelial cell; IgG = immunoglobulin G; LBPA = lyso(bis)phosphatidic acid; LPC = lysophosphatidylcholine; MCL = monolysocardiolipin; MS = mass spectrometry; PBS = phosphate-buffered saline; PBS-F = phosphate-buffered saline-foetal calf serum; PM = plasma membrane; SLE = systemic lupus erythematosus; TLC = thin-layer chromatography; TNF-α = tumour necrosis factor-alpha; VDAC-1 = voltage-dependent anion channel-1.

## Competing interests

The authors declare that they have no competing interests.

## Authors' contributions

CA carried out the ELISA experiments and the experiments on the endothelial cell line, participated in the design of the study and in the analysis of data, and drafted the manuscript. MS participated in the design of the study and in the analysis of data and helped to draft the manuscript. MB carried out the ELISA experiments and the experiments on endothelial cells and contributed to the interpretation of data. PC carried out the ELISA experiments and participated in the analysis of data. AL and TG carried out the immunoassay and participated in the analysis of data and in the revision of the manuscript. VM carried out the experiments of TLC immunostaining and immunofluorescence. FC performed the statistical analysis, the clinical associations, and the revision of the study. ME participated in the design of the study and in the revision of the manuscript. GV conceived of the study, participated in its design and coordination, and drafted the manuscript. All authors read and approved the final manuscript.

## Figures and Tables

**Table 1:** Demographic and clinical features of the patients studied

	APS	SLE
	(*n *= 18)	(*n *= 10)
Females/Males	15/3	9/1
Age in years		
Mean (range)	38.4 (28–68)	36.9 (18–59)
Disease duration in months		
Mean (range)	121 (1–322)	119 (12–300)
Vascular thrombosis		
Venous thrombosis (percentage)	14/18 (77.7)	0
Arterial thrombosis (percentage)	8/18 (44.4)	0
Recurrent thrombosis (percentage)	4/14 (28.5)	0
Pregnancy morbidity		
Normal foetus deaths (percentage)	0	0
Premature births (percentage)	1/18 (5.5)	0
Spontaneous abortions (percentage)	9/18 (50)	0
Serological features		
aCL IgG (percentage)	18/18 (100)	10/10 (100)
aCL IgM (percentage)	11/18 (61)	5/10 (50)
Anti-β_2_-GPI IgG	18/18 (100)	10/10 (100)
Anti-β_2_-GPI IgM	ND	ND
Lupus anticoagulant activity (percentage)^a^	15/18 (83.3)	5/10 (50)

^a^Lupus anticoagulant activity was deduced from recent clinical records. aCL, anticardiolipin antibody; APS, antiphospholipid syndrome; β_2_-GPI, beta_2_-glycoprotein I; IgG, immunoglobulin G; IgM, immunoglobulin M; ND, not done; SLE, systemic lupus erythematosus.
